# Siwu decoction mitigates radiation-induced immune senescence by attenuating hematopoietic damage

**DOI:** 10.1186/s13020-024-01036-3

**Published:** 2024-12-06

**Authors:** Mingyue Huang, Anping Ye, Haoyu Zhang, Yi Ru, Zhijie Bai, Yanyan Zhang, Yue Gao, Zengchun Ma

**Affiliations:** 1 Department of Pharmacology and Toxicology , Beijing Institute of Radiation Medicine, Beijing, China; 2 Department of Pharmaceutical Sciences, Jiangxi University of Traditional Chinese Medicine, Nanchang, Jiangxi China; 3 China Shineway Pharmaceutical Group Limited, Shijiazhuang, Hebei China

**Keywords:** Ionizing radiation, Senescence, Siwu decoction, Immune, HSPCs

## Abstract

**Background:**

To investigate the long term effects of ionizing radiation (IR) on hematopoietic stem/progenitor cells (HSPCs), immune tissues and cells, and the effects of Siwu decoction (SWD) on immune senescence mice.

**Methods:**

C57BL/6 J mice were exposed to 6.0 Gy ^60^Co γ irradiation. After 8-weeks of IR, SWD (5, 10, 20 g/kg/d) was administered for 30 days. The changes of HSPCs in bone marrow (BM) and T, B type lymphocyte and natural killer (NK) cells in spleen were detected by flow cytometry. The changes of peripheral blood cells were also examined. Hematoxylin–eosin staining were used to detect the pathological lesions of hippocampus, spleen and thymus tissues. Absolute mouse telomere length quantification qPCR assay kit was used to measure the telomere length of BM cells. The expression of factors associated with inflammation and aging such as p16, β-galactosidase in spleen, thymus and BM was determined.

**Results:**

Administration of SWD could increase the proportion of LSK (Lin−, Sca-1 + , c-Kit−), multipotent progenitor cells and multipotent progenitor cells and decrease the proportion of common myeloid progenitors and granulocyte–macrophage progenitors in BM. The proportion of B cells and NK cells in spleen and the content of white blood cells, red blood cells, hemoglobin, lymphocytes and eosinophils in peripheral blood were increased, at the same time, the proportion of neutrophils and monocytes was reduced by SWD. The pathological lesions of hippocampus, spleen and thymus were improved. The expression of p16 and β-galactosidase in spleen, thymus and BM was reduced and shortening of the telomere of BM cells was inhibited after administration. In addition, SWD could reduce the content of Janus activated kinase (JAK) 1, JAK2 and signal transducer and activator of transcription 3 (STAT3) in BM and spleen.

**Conclusions:**

SWD could slow down IR-induced immune senescence by improving hematopoietic and immunologic injury. SWD might reduce the inflammation level of BM hematopoietic microenvironment by acting on JAK/STAT signaling pathway, while the immune damage of mice was improved by affecting the differentiation of HSPCs. The remission of hematopoietic and immunologic senescence was further demonstrated at the overall level.

**Graphical Abstract:**

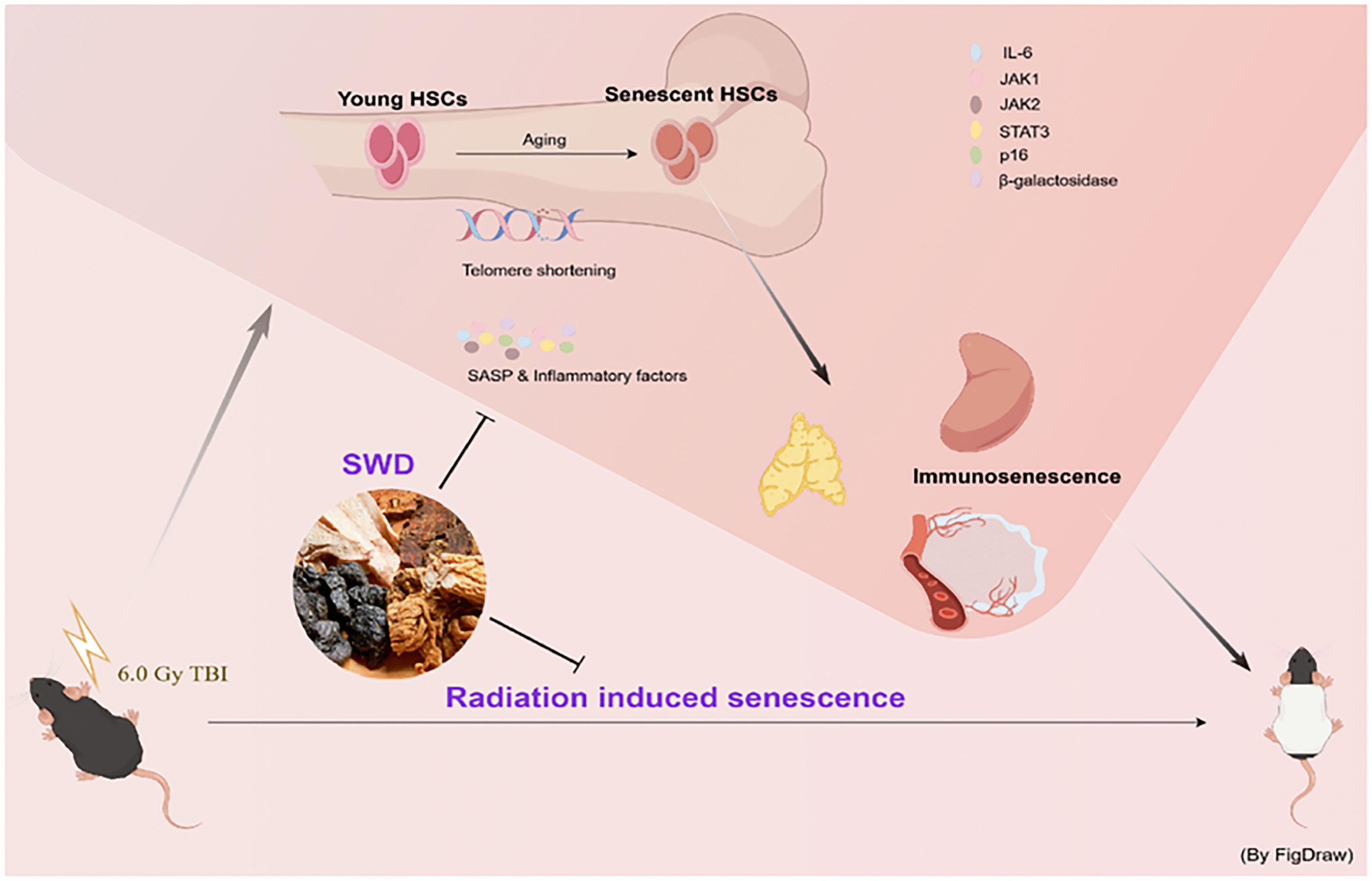

## Highlights

1. Siwu decoction (SWD) could relieve IR-induced senescence in mice.
2. SWD could improve the learning and memory ability and the pathological damage of hippocampus.
3. SWD could ameliorate the lymphatic/myeloid differentiation bias of bone marrow (BM) hematopoietic stem/progenitor cells (HSPCs) and immune imbalance in peripheral blood and spleen.
4. SWD could inhibit the shortening of telomere of BM cells.
5. SWD could alleviate the inflammation level of hematopoietic microenvironment and spleen, and reduce the expression of senescence related molecules (p16, β-galactosidase, etc.) in BM, spleen and thymus.

## Introduction

Global population aging is currently occurring at an unprecedented rate, leading to a demographic shift towards older individuals. This trend could have significant and far-reaching consequences as the later stages of life are associated with decreased adaptation and homeostasis mechanisms, resulting in heightened vulnerability to environmental or internal stress, disease, and mortality. Age-related disability and morbidity may have detrimental effects on human life quality, ultimately increasing the risk of death and causing challenges at individual, family, and community levels [1].

Aging mice models are usually treated with ionizing radiation (IR), galactose injection, and other methods, and the immediate cellular response to IR is characterized by the occurrence of γ-H_2_AX damage to DNA, as well as the activation and localization of DNA repair and other cellular mechanisms [2]. When the damage exceeds the normal cell repair capacity, these radiation-induced lesions either lead to senescence (i.e. permanent cell cycle arrest) or cell death, or persist and spread to future generations of cells. The body's hematopoietic and immunologic system are susceptible to radiation damage. During radiotherapy, the body's hematopoietic and immunologic functions are variably impaired. Radiation damages components within the hematopoietic stem cells (HSCs) niche, accelerates HSCs damage, and affects their self-renewal ability [3]. The dose-time relationship of radiation on the body results in damage to the hematopoietic system and suppression of immune function, with more severe effects as IR dose increases. Main manifestations include reduced quality of central or peripheral immune organs, decreased number of immunoactive cells, inhibited or disordered antibody formation, and abnormal regulation of cytokine networks [4]. The etiology and mechanisms of bone marrow (BM) HSCs injury encompass DNA damage, telomere shortening, heightened reactive oxygen species, epigenetic modifications, alterations in cell polarity, metabolic dysregulation, and changes in the hematopoietic microenvironment, which may further contribute to the aging of HSCs. The molecular pathways implicated primarily involve nuclear factor erythroid 2-related factor 2 (Nrf2) transcription factors, the Sirtuin family, Notch signaling pathway, Wnt/β-catenin pathway and p53/p21 signaling pathways [5, 6].

Research on the mechanisms and regulatory networks related to HSCs aging not only helps explain relevant theories of body aging and anti-aging but also holds significant importance for developing new strategies for BM-related therapy and identifying new drug targets. While there has been some progress in the development of chemical drugs with anti-aging effects, concerns remain regarding their potential side effects, specific targets, and the risk of multiple drug resistance. For instance, research conducted in the United States has shown that although rapamycin can extend the lifespan of mice by approximately 14%, its immunosuppressive effects may increase susceptibility to infectious diseases [7]. In contrast, traditional Chinese medicine (TCM) offers unique dialectical treatment systems and multi-target mechanisms to exert anti-aging effects with minimal adverse reactions. Studies have demonstrated that Rosa damascena extract can prolong the lifespan of Drosophila without negatively impacting reproduction or metabolic rate [8]. TCM is known for its focus on nourishing life and has garnered increasing attention for its anti-aging properties. While it may not be possible to prevent aging entirely, slowing down the process is certainly achievable.

Siwu decoction (SWD), renowned as the “leading prescription in gynecology”, consists of of *Rehmanniae Radix Praeparata*, *Angelicae Sinensis Radix*, *Paeoniae Radix Alba* and *Chuanxiong Rhizoma*. Clinical Chinese patent medicines, including Siwu Tablets and Siwu Mixture, were studied through activity-guided isolation research. It was discovered that the key active components responsible for the blood-tonifying effects of SWD are paeoniflorin, ferulic acid, and ligustrazine, among others. Notably, this research was the first to reveal the blood-tonifying activities of fructose and paeoniflorin [9]. Proteomics studies showed that SWD could restore the balance of disrupted protein groups associated with blood deficiency syndrome, primarily affecting functional proteins associated with the proliferation, differentiation, migration, and apoptosis of hematopoietic cells [10]. Metabolomics studies suggested that the therapeutic mechanism of SWD in treating blood deficiency syndrome may involve improving mitochondrial function, correcting disordered fatty acid β-oxidation, stabilizing cell membranes, maintaining osmotic pressure balance inside and outside the cells, regulating glycolysis, promoting normal energy metabolism, and enhancing antioxidant capacity and immunity [11]. Using UPLC-Q-TOF–MS/MS technology, a qualitative and quantitative analysis method was developed to study the drug-related chemical components in the serum of rats after a single administration of SWD. Several functional components of SWD, such as ferulic acid, paeoniflorin, and albiflorin, were identified in the medicated serum [12]. Futhermore, our previous work revealed the intervention mechanism of SWD on radiation-induced blood deficiency syndrome, mainly affecting hematopoietic stem/progenitor cells (HSPCs), apoptosis of BM cells, CD34 + cells, cell cycle, hematopoietic related genes and protein expression [9] and the pharmacodynamic material bases of SWD for anti-radiation are fructose, ferulic acid, paeoniflorin and ligustrazine [13]. In this research, we made a mouse model of immune senescence with a single dose of 6.0 Gy ^60^Co γ-ray exposure (dose rate 102.63 cGy/min) to investigate the effects of IR on HSPCs, immune tissues and cells, and the effects of SWD on immune senescence mice.

## Materials and methods

### Reagents

*Rehmanniae Radix Praeparata* (22070801, Henan, China), *Angelicae Sinensis Radix* (21061101, Gansu, China), *Paeoniae Radix Alba* (21122601, Anhui, China) and *Chuanxiong Rhizoma* (095210801, Sichuan, China) were purchased from Beijing Tongrentang Co.,ltd. Mouse catalase (CAT) (MM-44125M1), malondialdehyde (MDA) (MM-0897M1), beta-Nicotinamide adenine dinucleotide trihydrate (NAD +) (MM-1010M1), interleukin (IL)-6 (MM-0163M1), Janus activated kinase 1 (JAK1) (MM-46967M1), JAK2 (MM-46942M1), signal transducer and activator of transcription (STAT3) (MM-45741M1), cyclin-dependent kinase inhibitor 2A (CDKN2A/p16) (MM-44525M1) enzyme linked immunosorbent assay (ELISA) kits were purchased from Jiangsu Meimian Industrial Co., Ltd. Mouse taurine ELISA kit (JM-12220M2) was provided by Jiangsu Jingmei Biotech Co., Ltd. Bicinchoninic acid assay (BCA) protein quantitative kit (ZJ102) was from Shanghai Yaenzyme Biotechnology Co., Ltd. β-galactosidase microplate assay kit (ADS1062W) was purchased from Jiangsu Addison Biotechnology Co., Ltd. Hifair^®^ III 1st Strand cDNA Synthesis Super Mix for qPCR (11141ES60), Hieff UNICON^®^ Universal Blue qPCR SYBR Master Mix (11141ES) were from Yeasen Biotechnology (Shanghai) Co., Ltd. Animal tissue/cell genome DNA extraction kit (8028011) as well as Absolute Mouse Telomere Length Quantification qPCR Assay Kit (M8918, ScienCell^™^) purchased from akewe Biotech Co., Ltd. p16INK4a Polyclonal Antibody (PA5-20379) and beta Galactosidase Polyclonal Antibody (PA5-102503) were from Invitrogen (US). Antibodies used in flow cytometry were listed in Table 1.

**Table 1:** Antibodies used in flow cytometry

Antibody	Clone	Conjugate	Source	LOT
Hematopoietic lin cocktail	–	EF450	eBioscience	88–7772–72
CD117	ACK2	PE-Cy7	eBioscience	25–1172–82
LY-6/E	D7	PE	eBioscience	12–5981–82
CD34	RMA34	FITC	eBioscience	11–0341–85
CD127	A7R34	APC	eBioscience	17–1271–82
CD48	HM48-1	APC-EF780	eBioscience	47–0481–82
CD150	TC15-12F12.2	BV786	Biolegend	115937
CD16/32	S17011E	BV510	Biolegend	101333
CD19	HIB19	PE	eBioscience	12–0199–42
CD45R	RA3-6B2	EF506	eBioscience	69–0452–82
CD3e	145-2C11	PE-Cy7	eBioscience	25–0031–82
CD4	GK1.5	APC-EF780	eBioscience	47–0041–82
CD8a	53–6.7	APC	eBioscience	17–0081–82
NK1.1	PK136	FITC	eBioscience	11–5941–82
7-AAD	–	–	Biolegend	420404

### Animals and IR

According to the *Taiping Huimin Hejiju Prescription*, the composition of SWD includes 15 g of *Rehmanniae Radix Praeparata*, 10 g of *Angelicae Sinensis Radix*, 10 g of *Paeoniae Radix Alba*, and 6 g of *Chuanxiong Rhizoma*, totaling 41 g per day. The equivalent dose ratio between mice and humans is 9.1 [14]. Using the standard human body weight of 70 kg for conversion, the equivalent dose of SWD for mice can be calculated as follows:

$${\text{Equivalent dose of SWD for mice }} = \, \left( {{41 }/{ 7}0} \right) \, *{ 9}.{\text{1 g}}/{\text{kg}}/{\text{d }} = {\text{ 5 g}}/{\text{kg}}/{\text{d}}$$

SPF C57BL/6 J male mice, 21–24 g, were purchased from Beijing Weitong Lihua Laboratory Animal Technology Co., Ltd. The mice were raised in the Animal Center of the Academy of Military Medical Sciences (AMMS) and randomly divided into 5 groups: normal control group (NC), irradiation group (Model), low-dose SWD administration group (SWD-L, 5 g/kg/d i.g.), medium-dose SWD administration group (SWD-M, 10 g/kg/d i.g.), high-dose SWD administration group (SWD-H, 20 g/kg/d i.g.), which equivalent to 1, 2, 4 times the clinical equivalent dose (drawing from our two decades of research experience on SWD, which is most appropriate), with 7 mice in each group. After 7 days of adaptive feeding, the mice, except for NC, were exposed to 6.0 Gy ^60^Co γ-ray at a dose rate of 102.63 cGy/min to induce hematopoietic-immune injury. The mice received a continuous administration of drugs for 30 days 8 weeks after irradiation. The experiment was performed in accordance with the guidelines of the European Community and approved by the Institution of animal Care and Use Committee of AMMS: IACUC-DWZX-2023–547.

### Decocting and preparation of SWD

410 g of SWD (*Rehmanniae Radix Praeparata* 150 g, *Angelicae Sinensis Radix* 100 g, *Paeoniae Radix Alba* 100 g, *Chuanxiong Rhizoma* 60 g) was added to Soxhlet extraction device, soaked in 3 L of ultra-pure water overnight, steamed 3 times the next day, 1.5 h each time, combined with the decoction, concentrated, poured into the freeze dryer. The freeze-dried powder of SWD was prepared by cryogenic freeze-drying for 35 h. At last, the total of 410 g of raw herbs yielded 221.7511 g of freeze-dried powder, with a yield rate of 54%. The corresponding concentration of drug was prepared by freeze-dried powder and ultra-pure water.

### Mice behavioral experiment

**Open field test (OFT):** The experimental animal is quickly placed in the central area of the experimental box and immediately left, and the animal behavior analysis software is opened 2 min later to automatically record the activities of the animal in the box for 5 min.

**Grasp force measurement:** Place the grasp force tester in a horizontal position and position the mouse on it. Grasp the tail of the mouse and gently apply even force to pull back, causing the mouse to release its claw. Record the maximum grasp force achieved. Each mouse was measured 3 times, and the maximum value was used to evaluate the muscle strength of the mouse.

**Treadmill stress test:** First at the speed of 10 m/min (5 min) and 20 m/min (5 min) adaptive training 10 min, and then 30 m/min until the mice exhaustion. Runway angle was set 15°, current 0.5 mA. It was considered as exhaustion while the mice were shocked 5 times consecutively within 2 min.

**Novel-object preference test (NOP):** On the first day, two identical objects were placed in the apparatus, and mice were allowed to freely explore for 10 min. On the second day, one of the identical objects was replaced with a different object in the apparatus, and the mice were also allowed to explore for 5 min. The duration of exploration for each object was measured. The recognition index (RI) is calculated as follows: RI = T2/(T1 + T2) × 100%, where T1 is the time for mice to explore familiar objects and T2 is the time to explore new objects.

### Blood cell counts

At the end of the administration, blood samples were collected and a hematology analyzer (Sysmex XN-1000 V) was used to determine blood cell counts. The content of white blood cells (WBC), red blood cells (RBC), hemoglobin (HGB), and the proportion of neutrophils (NEUT%), lymphocytes (LYMPH%), eosinophils (EO%) and monocytes (MONO%) were counted.

### Calculation of organ index

The mice in each group were weighed, and the spleen and thymus tissues were removed and weighed after euthanasia.

$${\text{Organ index}} = {\text{organ weight }}\left( {{\text{mg}}} \right)/{\text{body weight }}\left( {\text{g}} \right)$$

### Flow cytometry

Single cell samples from the BM and spleen were labeled with corresponding fluorescent antibodies to detect mouse HSPCs as well as immune cells. The primary HSPCs in mice, including lymphoid and myeloid progenitors, were identified based on the expression of LIN, Sca-1, c-kit, CD34, CD16/32, and CD127. T cells were characterized by the presence of CD3, CD4, and CD8, while B cells were identified using CD19 and B220. Natural killer (NK) cells were distinguished by the expression of NK1.1 [15]. This approach was utilized to assess the impact of IR on the composition of hematopoietic and immune cell populations over time.

### ELISA

BM and spleen proteins were quantified in which the contents of IL6, JAK1, JAK2, STAT3 and p16 proteins were detected by ELISA kits. All operations were carried out according to the manufacturer’s instructions, through the steps of sample addition, enzyme addition, warming, washing, color development, termination and finally the absorbance is measured in sequence at 450 nm.

### Detection of the activity of β-galactosidase

The BM samples were adjusted to the same concentration level according to the results of protein quantification. The temperature and wavelength of the microplate reader were set at 37 ℃ and 405 nm. Each measuring tube (A_1_) was provided with a contrast one (A_0_), ΔA = A_1_-A_0_.

### Quantitative real-time PCR (qPCR)

Total RNA from mice BM was extracted and reverse-transcribed into cDNA. The mRNA levels of different genes were quantitatively analyzed by 2^−△△Ct^ using β-actin as the internal reference. Primers p21, GLB1, Socs3 and β-actin were synthesized in Beijing Tianyi Huiyuan Biotechnology Co., Ltd. The sequences are shown in Table 2.

**Table 2:** Primer sequences

Gene	Forward primer (5′-3′)	Reverse primer (5′-3′)
β-actin	CCTCACTGTCCACCTTCCA	GGGTGTAAAACGCAGCTCA
p21	CCTGGTGATGTCCGACCTG	CCATGAGCGCATCGCAATC
GLB1	GGATGGACAGCCATTCCGAT	CAGGGCACGTACATCTGGATA
Socs3	GACCAAGAACCTACGCATCCA	CGCCCCCAGAATAGATGTAGTA

### Determination of telomere length

Genomic DNA of mice BM cells was extracted and two qPCR reaction systems were prepared, one was telomere primer stock and the other was single copy reference gene primer stock, and the results were calculated by 2^−△△Ct^ method.

### Histological analysis

The brain, thymus and spleen tissues of mice (n = 3) were fixed with 4% paraformaldehyde, dehydrated and embedded with paraffin to prepare paraffin sections with a thickness of about 5 µm.

**Hematoxylin–eosin staining (H&E):** Sections were placed in hematoxylin staining solution, soaked for 10–15 min, washed with distilled water and then added to eosin dyeing solution for 15 s and washed with distilled water. After which, the sections were dehydrated with 75, 85, 95 and 100% ethanol gradient for 2 min, respectively and were sealed with xylene transparent and neutral glue.

**Immunofluorescence staining (IF):** The fluorescence intensity of p16 protein and β-galactosidase in spleen and thymus of mice was detected through the steps of permeability, sealing, antibody incubation, etc. Image J 2.0 was used to calculate the mean density of fluorescence.

### Statistical analyses

All the data in this experiment were repeated for more than 3 times, and the data were represented by mean ± standard deviation ($\overline{x}$± s). GraphPad Prism 8 was used to plot the data. One-way ANOVA was used to analyze the data between multiple groups, and t test was used to analyze the data between two groups. *p* < 0.05 was considered significant.

## Results

### SWD alleviated IR-induced senescence in mice

**Fig. 1:**
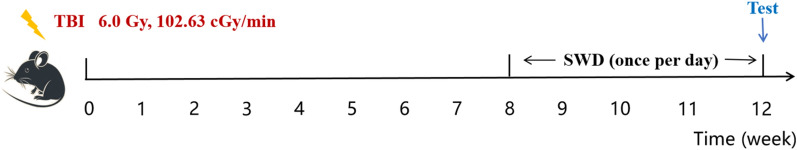
Schematic of the experimental procedure for mice experiencing IR and SWD intervention

**Fig. 2:**
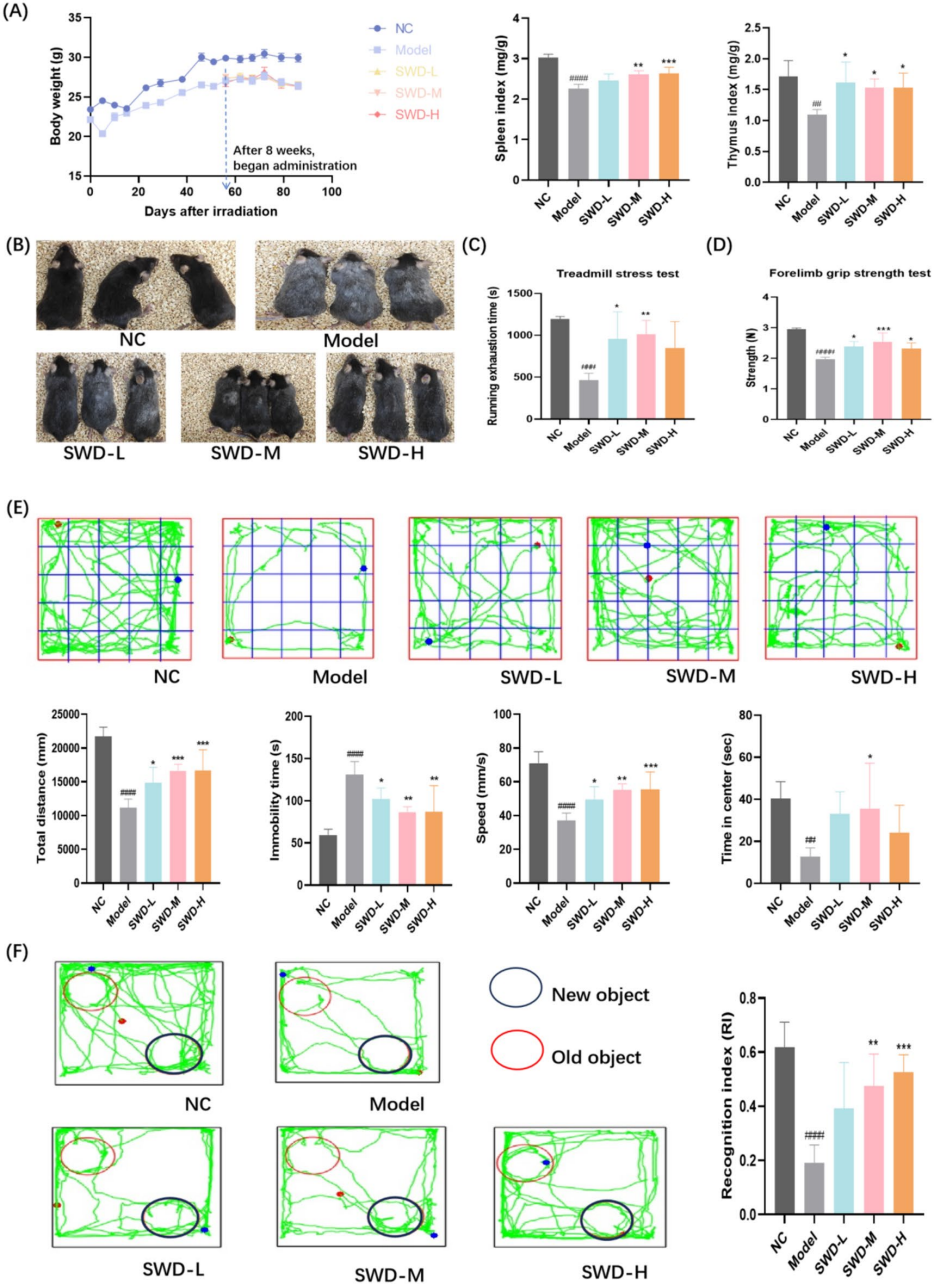
SWD relieved IR-induced senescence in mice. **A** Body weight, spleen index and thymus index (n = 6); **B** Snapshot comparison of mice from each group respectively (n = 3); **C** Running exhaustion time (n = 5); **D** Grip strength (n = 5); **E** Representative trace in OFT of each group of mice and total travel distance, immobility time, speed as well as time of entry into the central area in OFT (n = 6); **F** Representative trace and RI of each group of mice in NOP. RI = T2/(T1 + T2) × 100%, where T1 is the time spent exploring familiar objects and T2 is the time spent exploring new objects (n = 5). ^##^*p* < 0.01, ^###^*p* < 0.001, ^####^*p* < 0.0001 versus the control group; *p < 0.05, **p < 0.01, ***p < 0.001 versus the model group ($\overline{x}$ ± s)

The irradiated mice were randomly assigned to groups and numbered on the 50th day post-irradiation, and received a continuous administration of drugs for 30 days 8 weeks after irradiation (Fig. 1). As depicted in Fig. 2A, both medium and high doses of SWD demonstrated a significant increase in the spleen index. Additionally, SWD at low, medium, and high doses exhibited a significant increase in the thymus index of irradiated mice. After 12 weeks of exposure to IR, it was observed that the hair of C57 mice turned gary and lost luster, their running endurance decreased, and their grip strength weakened (Fig. 2B–D), indicating an senescence phenotype, which were improved by the treatment with SWD. Additionally, the activity of mice in the model group in the open field was significantly reduced (Fig. 2E). This reduction manifested as decreased activity distance, slowed moving speed, reduced time spent in the central area, and prolonged rest time. SWD was found to increase both the distance and activity level of mice, with the medium dose of the drug increasing the time that irradiated mice spent in the central region. Failure to distinguish between new and old objects in NOP may indicate issues with memory or cognition related to aging, brain damage, etc. [16]. It could be seen that the recognition index (RI) of model group mice decreased, but administration of medium and high doses of SWD significantly extended exploration time for the new object (Fig. 2F). Overall, SWD appeared to decelerate the aging process in irradiated mice, with the medium dose (10 g/kg/d) showing the most promising effect.

### SWD reduced the level of oxidative stress in mice and the pathological damage of hippocampus

**Fig. 3:**
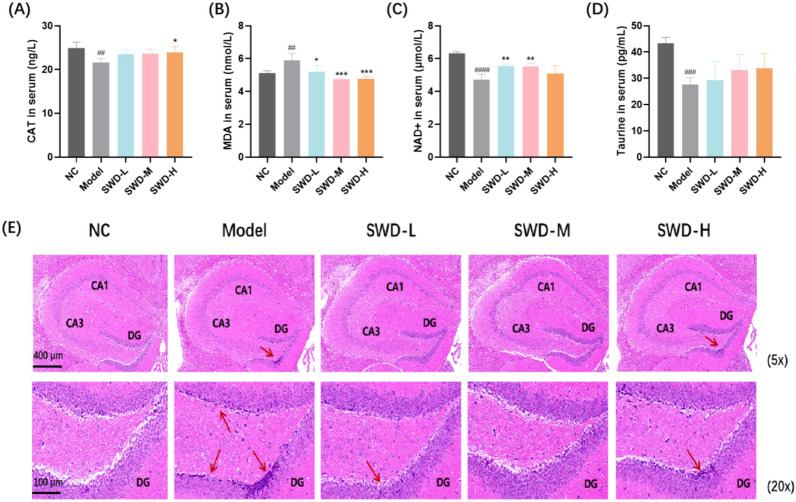
SWD reduced the level of oxidative stress in mice serum and the pathological damage of hippocampus. The content of (**A**) CAT; **B** MDA; **C** NAD + ; **D** Taurine in mice serum. ^##^*p* < 0.01, ^###^*p* < 0.001, ^####^*p* < 0.0001 versus the control group; *p < 0.05, **p < 0.01, ***p < 0.001 versus the model group (n = 5, $\overline{x}$ ± s); **E** H&E staining results of hippocampus in each group (n = 3). Mice exposed to IR exhibited hippocampal abnormalities, including intensified cell staining in the DG region, indistinct nuclear and cytoplasmic boundaries, and markedly decreased white matter (red arrow)

SWD was found to enhance the antioxidant capacity of irradiated mice, with high doses increasing serum CAT content and all three doses significantly reducing MDA levels. Additionally, low and medium doses of SWD were shown to increase NAD + content in the serum (Fig. 3A–C). Furthermore, research has indicated that taurine deficiency is a key factor in the aging process [17]. Therefore, we measured the serum taurine levels in mice. As shown in Fig. 3D, there was a significant decrease in serum taurine levels in the model group, which could be partially restored by SWD treatment. H&E staining was conducted on the hippocampus of mice in each group. It was observed that after 12 weeks of irradiation, the hippocampus of mice in the model group exhibited abnormalities, with deepened cell staining in the dentate gyrus (DG) region, unclear boundaries between nucleus and cytoplasm, and a significant reduction in white matter (Fig. 3E). The hippocampus is a crucial brain region for memory formation, with the DG region playing a key role in this process [18, 19]. Hippocampal white matter, a protein located on the surface of nerve cells, is essential for normal neural activities in the hippocampus [20]. In the low dose SWD administration group, there was a noticeable enhancement in cell staining depth but a significant reduction in white matter. In the high dose group, some cells still displayed deep staining, while the medium dose demonstrated the most substantial improvement.

### SWD regulated the imbalance of lymphoid-myeloid differentiation

**Fig. 4:**
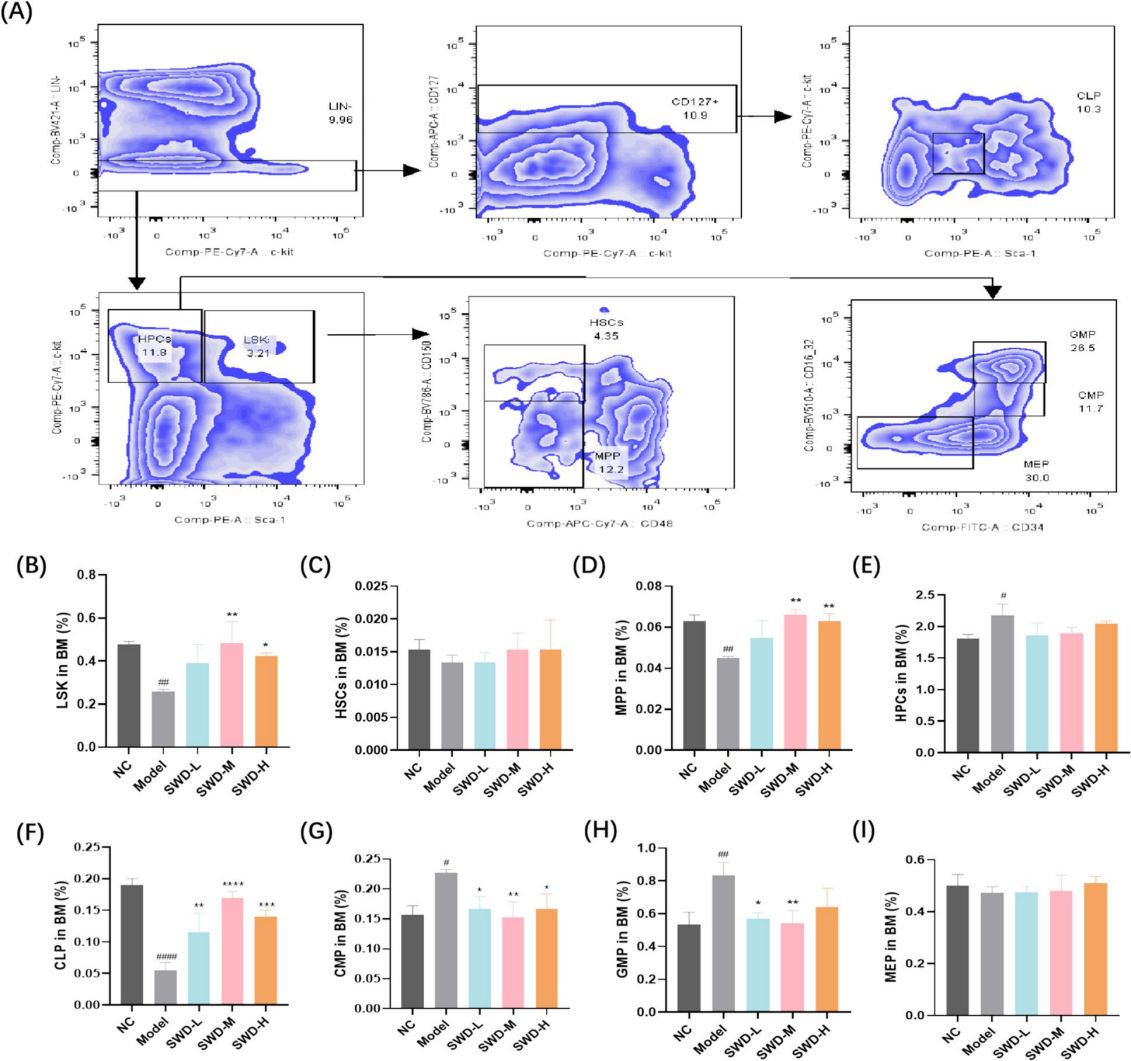
SWD regulated the imbalance of lymphoid-myeloid differentiation. **A** Flow cytometry gating strategy; The proportion of (**B**) LSK; **C** HSCs; **D** MPP; **E** Hematopoietic progenitor cells (HPCs); **F** CLP; **G** CMP; **H** GMP; **I** Megakaryocytic-erythroid progenitors (MEP) in BM. ^#^*p* < 0.05, ^##^*p* < 0.01, ^####^*p* < 0.0001 versus the control group; *p < 0.05, **p < 0.01, ***p < 0.001, ****p < 0.0001 versus the model group (n = 3, $\overline{x}$ ± s)

After 12 weeks of exposure to 6.0 Gy IR, the proportion of LSK (Lin−, Sca-1 + , c-Kit −) cells in BM of C57 mice did not recover (Fig. 4B), and multipotent progenitor cells (MPP) were largely reduced (Fig. 4D). Medium and high doses of SWD could increase the proportion of these two groups. Furthermore, the model group of mice exhibited a decrease in lymphoid progenitors and an increase in myeloid progenitors. Administration of three doses of SWD resulted in a significant increase in the proportion of common lymphoid progenitors (CLP) and a decrease in the proportion of common myeloid progenitors (CMP) in BM (Fig. 4F–G). The low and medium doses were able to reduce the proportion of granulocyte–macrophage progenitors (GMP) (Fig. 4H). CLP were the precursors of T cells, B cells, and NK cells; GMP were the progenitors of MONO and granulocytes. Consequently, we assessed the proportions of T cells, B cells, and NK cells in the spleen of the mice, as well as MONO%, EO%, NEUT% and LYMPH% in the peripheral blood, which were presented below.

### SWD ameliorated the immune imbalance of peripheral blood and spleen

**Fig. 5:**
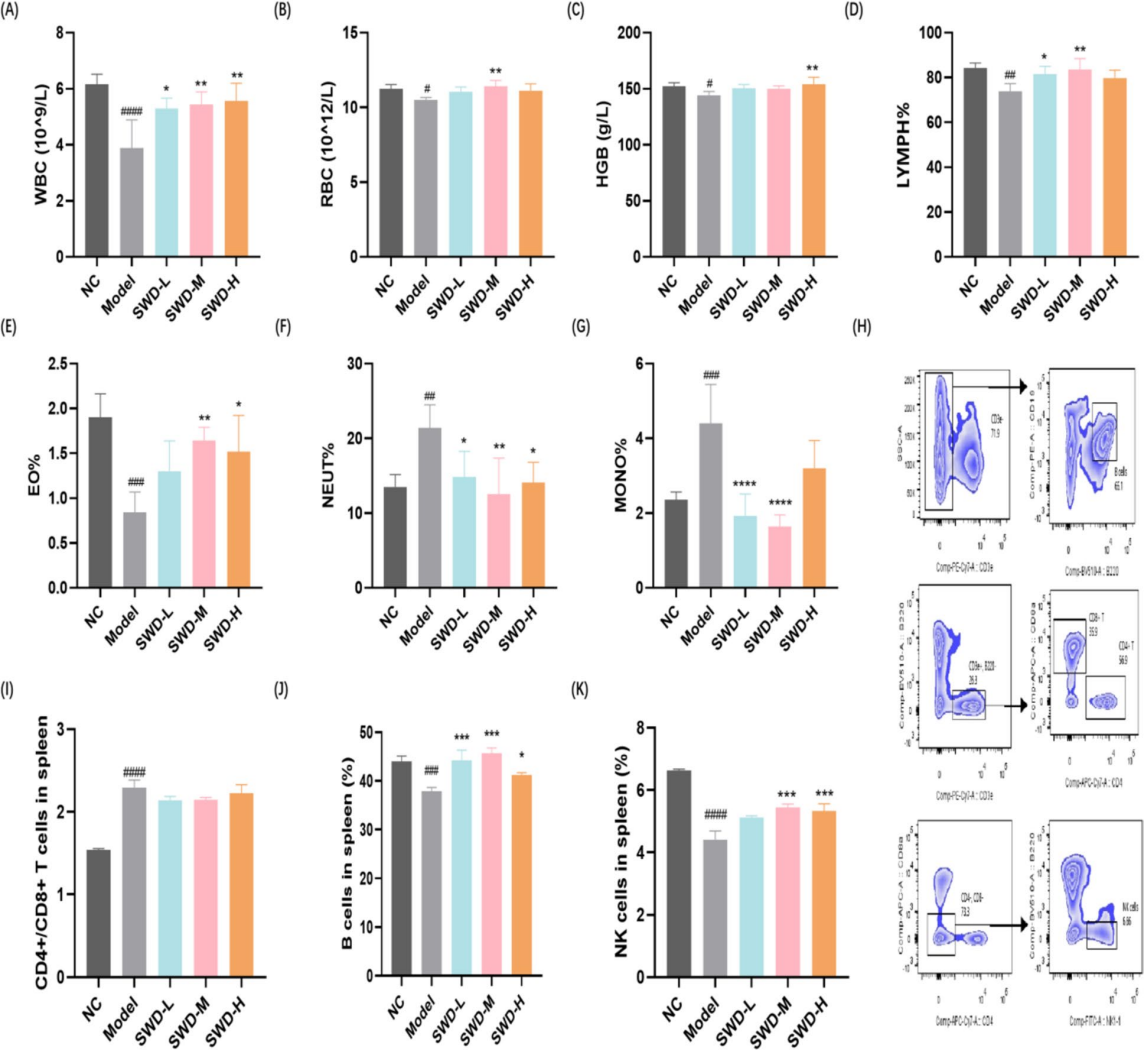
SWD ameliorated the immune imbalance of peripheral blood and spleen. The content of (**A**) WBC; **B** RBC; **C** HGB; **D** LYMPH%; **E** EO%; **F** NEUT%; **G** MONO% in peripheral blood (**A**–**G**, n = 5); **H** Flow cytometry gating strategy for splenic immune cells; **I** CD4 + /CD8 + T cells; **J** B cells; **K** NK cells in the spleen (**I**–**K**, n = 3). ^#^*p* < 0.05, ^##^*p* < 0.01, ^###^*p* < 0.001, ^####^*p* < 0.0001 versus the control group; *p < 0.05, **p < 0.01, ***p < 0.001, ****p < 0.0001 versus the model group ($\overline{x}$ ± s)

Based on the findings presented in Fig. 4, it could be concluded that SWD had the potential to mitigate inflammation and immune damage in irradiated mice. All three doses of the drug were found to increase the number of WBC, decrease NEUT%, and elevate the proportion of B cells in the spleen (Fig. 5A, F, J). Additionally, the medium dose was observed to significantly increase RBC (Fig. 5B), while a high dose of SWD led to an increase in HGB levels (Fig. 5C). Furthermore, low and medium doses of SWD were shown to enhance LYMPH% and reduce MONO% (Fig. 5D, G). The proportion of EO in peripheral blood and NK cells in spleen were also significantly increased by medium and high doses of SWD (Fig. 5E, K).

### SWD reduced the expression of inflammatory/aging related factors in BM

**Fig. 6:**
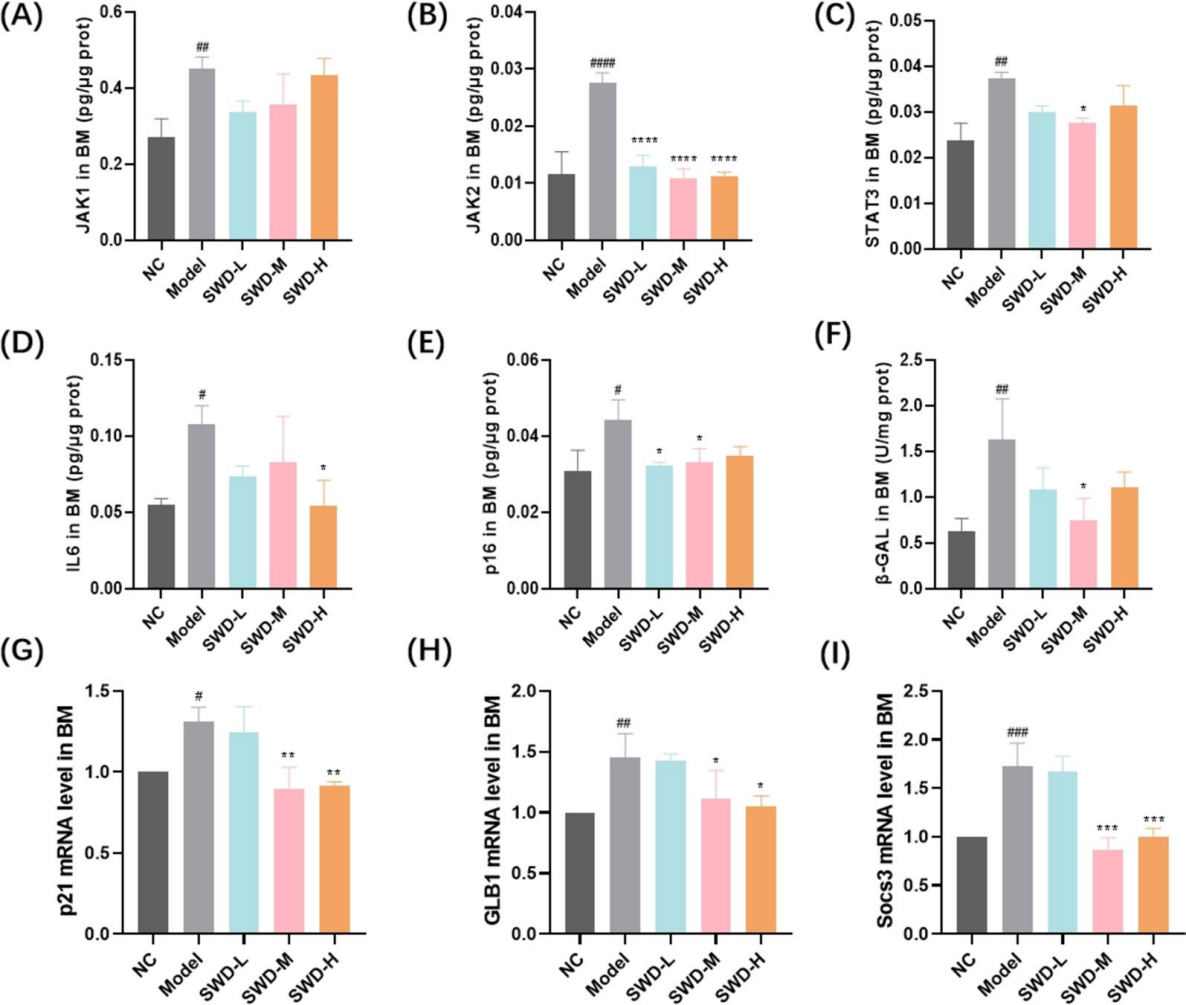
SWD reduced the expression of inflammatory/aging related factors in BM. The protein ratio (the content of protein/total protein concentration) of (**A**) JAK1; **B** JAK2; **C** STAT3; **D** IL-6; **E** p16; **F** The activity of β-galactosidase; The expression level of (**G**) p21; **H** GLB1; **I** Socs3 in BM. ^#^*p* < 0.05, ^##^*p* < 0.01, ^###^*p* < 0.001, ^####^*p* < 0.0001 versus the control group; *p < 0.05, **p < 0.01, ***p < 0.001, ****p < 0.0001 versus the model group (n = 3, $\overline{x}$ ± s)

IR had been reported to promote cellular senescence, with high expression of age-related β-galactosidase (SA-β-gal) and age-specific genes (p16, p12, and Bcl-2) in irradiated bone marine-derived macrophages [21]. It was observed that the expression of JAK1, JAK2, STAT3 and p16 proteins in BM of irradiated mice was significantly elevated, along with an increase in β-galactosidase activity. Additionally, there was a notable increase in the level of pro-inflammatory factor IL-6. Treatment with three doses of SWD notably reduced the content of JAK2 protein; low and medium doses of SWD decreased the secretion of p16 protein; medium dose administration led to a decrease in the content of STAT3 protein and the activity of β-galactosidase, while high dose treatment resulted in decreased IL-6 expression (Fig. 6A–F). In the model group, GLB1 and Socs3 genes exhibited high expression in mice BM cells, while SWD at medium and high doses significantly decreased their expression (Fig. 6G–I). These suggested that SWD may attenuate BM microenvironment inflammation induced by IR, potentially through modulation of the JAK/STAT signaling pathway.

### SWD inhibited the telomere shortening of BM cells

**Fig. 7:**
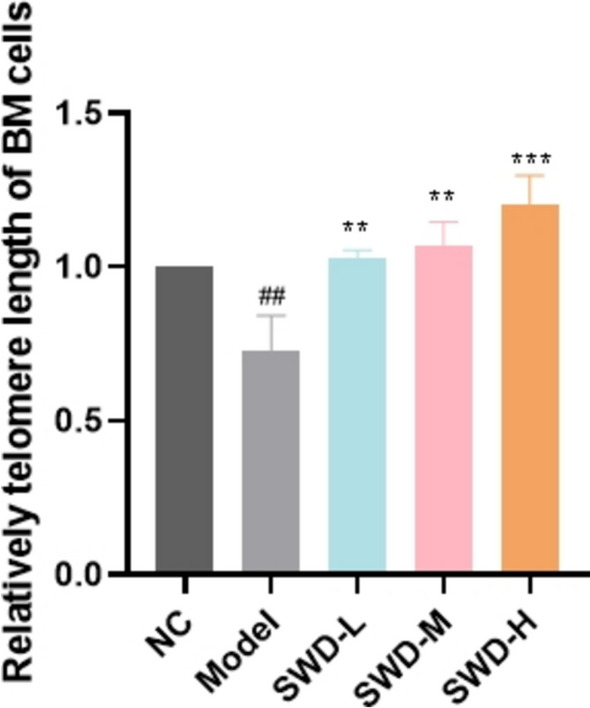
SWD inhibited the telomere shortening of BM cells. ^##^*p* < 0.01 versus the control group; **p < 0.01, ***p < 0.001 versus the model group (n = 3, $\overline{x}$ ± s)

Telomere depletion was a characteristic of cellular senescence. In this study, the telomeres of BM cells in the model group were found to be shortened compared to the control group, and SWD administration was shown to significantly inhibit this process, which suggested that SWD might play a role in anti-aging by preventing telomere shortening (Fig. 7).

### SWD reduced the pathological damage of spleen and thymus

**Fig. 8:**
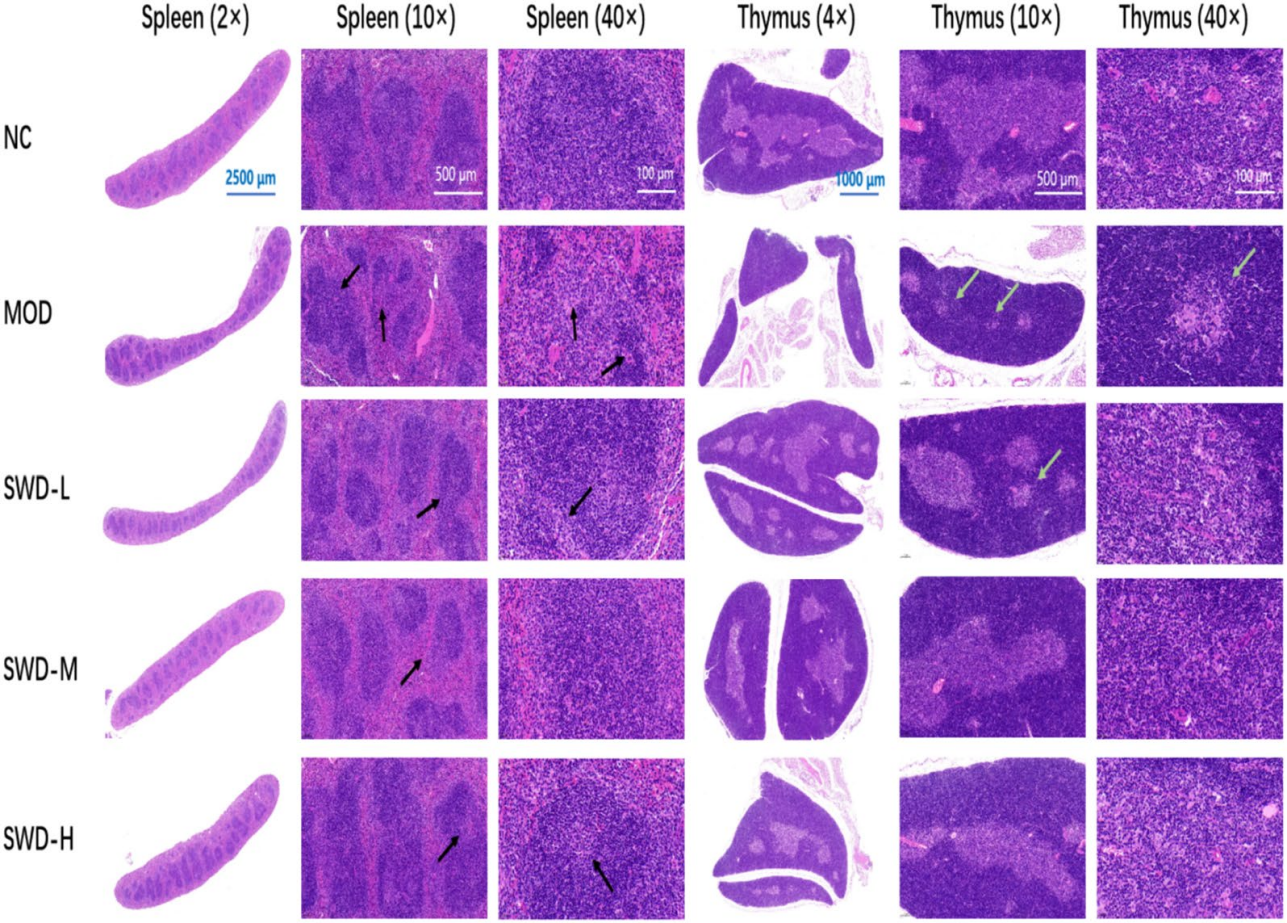
H&E staining results of spleen and thymus in each group. The spleen and thymus of irradiated mice were atrophied, the spleen bodies were significantly reduced, and the volume of lymph nodules became smaller (black arrow). The thymus medulla was prominently reduced, and the number of thymus corpuscles and lymphocytes was decreased (green arrow)

Under the same visual field, irradiated mice exhibited notable thymic atrophy, decreased medulla, and reduced numbers of thymic corpuscles and lymphocytes. There was also a significant decrease in splenic corpuscles and lymph nodule volume. SWD demonstrated a significant improvement in enhancing the spleen and thymus medulla, increasing the number of lymphocytes and thymus volume of irradiated mice (Fig. 8).

### SWD reduced the expression of aging related factors in spleen and thymus

**Fig. 9:**
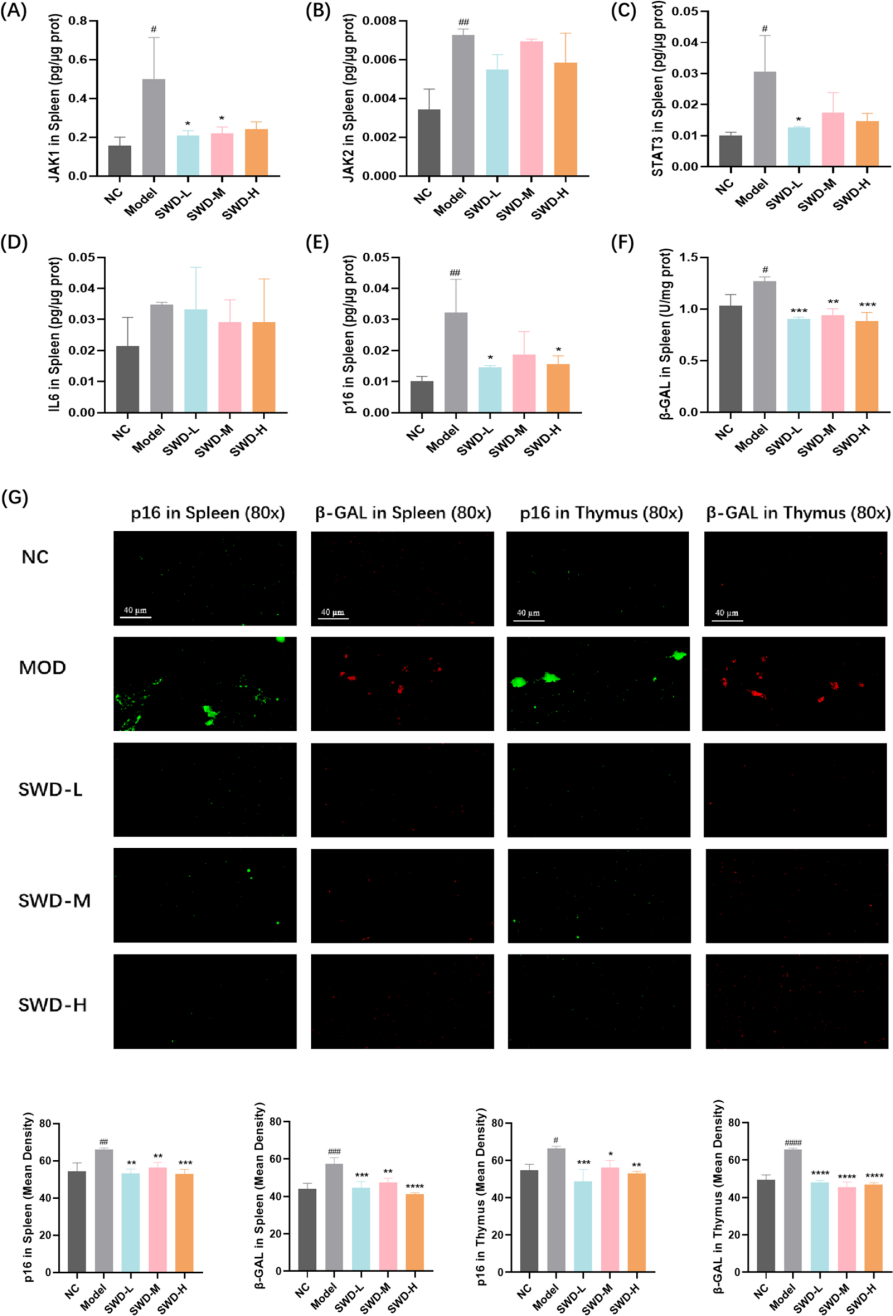
SWD reduced the expression of aging related factors in spleen and thymus. The protein ratio (the content of protein/total protein concentration) of (**A**) JAK1; **B** JAK2; **C** STAT3; **D** IL-6; **E** p16; **F** The activity of β-galactosidase in spleen; **G** Representative images and semi-quantitative analysis of p16 (green) and β-galactosidase (red) in spleen and thymus in each group. At least three 40-fold fields of view were randomly selected for each slice in each group. When capturing images, it was ensured that the background lighting remained consistent across all photos. The Image J 2.0 software was utilized to convert the green/red fluorescent monochrome images into black and white, with a standardized selection of black for fluorescence assessment. The integrated optical density (IOD) and pixel AREA of the tissue were measured for each image, and the mean density value IOD/AREA was calculated. ^#^*p* < 0.05, ^##^*p* < 0.01, ^###^*p* < 0.001, ^####^*p* < 0.0001 versus the control group; *p < 0.05, **p < 0.01, ***p < 0.001, ****p < 0.0001 versus the model group (n = 3, $\overline{x}$ ± s)

Consistent with the results from BM cells, the levels of JAK1, JAK2, STAT3 and p16 proteins, as well as the activity of β-galactosidase in the spleen of irradiated mice, were significantly increased. Administration of low and medium doses of SWD resulted in reduced levels of JAK1 (Fig. 9A); low dose administration led to decreased STAT3 levels (Fig. 9C); low and high doses of SWD resulted in reduced levels of p16 (Fig. 9E); all three doses of SWD led to a reduction in the activity of β-galactosidase in the spleen (Fig. 9F). In addition, IF was also utilized to assess the expression of p16 protein and β-galactosidase in the spleen and thymus of mice in each group. It was evident that the model group exhibited elevated expression of these two proteins, which significantly decreased following administration of SWD (Fig. 9G). Semi-quantitative analysis results indicated that three doses of SWD were able to reduce the expression of p16 protein and β-galactosidase in both spleen and thymus, suggesting that SWD could mitigate IR-induced immune senescence.

## Discussion

The irradiated mice exhibited signs of premature senescence, including early onset frailty, reduced cognitive function, and higher mortality rates as documented [22]. However, there is a limited amount of research on IR-induced premature aging, and very few corresponding measures have been developed to date [23]. Numerous traditional Chinese herbs have demonstrated significant benefits in suppressing tumor progression, enhancing radiotherapy sensitivity, boosting the immune system's function, and mitigating the damage caused by radiotherapy. For example, *Angelicae Sinensis radix* and its active components exhibit potent anticancer effects in liver cancer, oral cancer, and lung cancer by inducing apoptosis, reducing multidrug resistance, or modulating lymphocyte activity to strengthen immunity [24, 25]. Moreover, decoctions of *Angelicae Sinensis radix* can increase the radiation sensitivity of human liver cancer cells by regulating caspase-dependent apoptotic proteins [26]. Studies have demonstrated that the inhibitory effect of ferulic acid on IR was mediated through the enhancement of the antioxidant defense system in BM injury, loss of bone mass and senescence of HSCs [27]. *Angelica sinensis radix* is one of the components in SWD, and ferulic acid is one of its primary active ingredients. Research has shown that Taohong Siwu decocotion can assist radiotherapy in reducing angiogenesis and lymphangiogenesis in the treatment of breast cancer, while also alleviating the bone marrow suppression side effects induced by radiotherapy drugs [28]. In this study, we assessed the therapeutic effects of SWD on the hematopoietic system of prematurely aged mice and investigated the underlying mechanisms. Our findings indicated that SWD improved the aging phenotypes in our mouse model. Additionally, SWD treatment mitigated immune senescence and BM microenvironment inflammation possibly by suppressing the JAK/STAT signaling pathway.

The therapeutic effects of SWD on IR-induced aging phenotypes were initially observed. The results demonstrated that abnormal body appearance, decreased muscle strength, degenerated exercise capacity, and impaired memory function were mitigated by the administration of SWD. The antioxidant effect of SWD was subsequently evaluated in mice. MDA served as a biomarker for lipid oxidative damage [29]. CAT was typically phosphorylated and activated by protein kinase in order to maintain hydrogen peroxide (H_2_O_2_) homeostasis and safeguard cells from oxidative stress [30]. NAD + , a coenzyme involved in redox reactions, played a central role in energy metabolism and could directly and indirectly impact numerous crucial cellular functions, including metabolic pathways, DNA repair, chromatin remodeling, cell aging, and immune cell function. These cellular processes and functions were vital for the maintenance of tissue and metabolic homeostasis as well as for healthy aging [31]. Our findings demonstrated that SWD significantly enhanced the serum levels of CAT and NAD + in mice, while reducing MDA content, which suggested that SWD possessed medicinal properties capable of augmenting the antioxidant capacity in IR-induced premature senescence mice.

Hematopoiesis is an ongoing process during which blood cells are generated, maintained at normal levels, and increased in response to demand [32]. Some researchers have found that whole-body IR selectively induces HSCs senescence, with a significant reduction in MPP being one of the characteristics [33, 34]. Furthermore, the decrease in lymphoid progenitors and increase in myeloid progenitors are features of immune senescence with peripheral blood cell counts reflecting immune and inflammatory processes in vivo [35, 36]. We observed hematopoietic cells in the BM and noted an increased proportion of myeloid progenitor cells and a decreased proportion of MPP and lymphoid progenitor cells, indicating a bias towards the myeloid lineage of HSCs in IR-induced premature senescence mice. Subsequently, peripheral blood cells and spleen immune cells were detected. Significant reductions in WBC, RBC, HGB, LYMPH%, and EO% in the peripheral blood indicated impaired immune function [37]. Meanwhile, notably increased NEUT% and MONO% suggested the presence of inflammation [38]. In the spleen, there was a significant increase in the proportion of CD4 + /CD8 + T cells due to high doses of radiation activating the peripheral immune system [39]. However, the decrease in B cell and NK cell proportions also indicated a decline in immune function, pointing to immune impairment in mice. The administration of three doses of SWD was found to effectively modulate immune imbalance in the peripheral blood and spleen of irradiated mice, with the medium dose (10 g/kg/d) demonstrating the most favorable outcome. These findings indicate that SWD has the potential to influence the differentiation of HSPCs, subsequently impacting downstream blood and immune cells, thereby mitigating inflammation and immune damage caused by IR. In conclusion, SWD demonstrated a significant inhibitory effect on the IR-induced increase in myeloid cells and could improve the skew towards myeloid lineage in mice.

An increasing number of findings indicate that certain active components and formulations of TCM can exert a distinct anti-aging effect by enhancing telomerase activity or inhibiting telomere shortening. Research results have demonstrated that *Angelica sinensis* polysaccharides could significantly enhance the learning and memory capabilities of mice, down-regulate p53 expression in HSCs, and increase telomere length and telomerase activity [40]. Similarly, in our study, we observed a significant reduction in telomere length of BM cells in the model group after 12 weeks of IR. However, this telomere shortening was effectively attenuated by treatment with SWD. CDKN2A, also known as p16, and β-galactosidase are frequently utilized as indicators of cellular senescence [41–43]. The p21 gene, a recent discovery, is an important member of the cyclin-dependent kinase inhibitor family. In addition to its role in maintaining cell cycle arrest in senescent cells, p21 has been found to play a significant role in establishing an age-related secretory phenotype (SASP) through RB-dependent transcription of selected SMAD and STAT transcription factors [44]. Results demonstrated that SWD exhibited a significant reduction in the expression of p16 protein and β-galactosidase in mice BM, spleen, and thymus. Additionally, it effectively suppressed p21 gene expression in BM.

IR-induced hematopoietic damage disrupts the dynamic balance of immune and blood cells, thereby exacerbating the body’s inflammatory response. Our previous work has demonstrated that paeoniflorin is one of the main components in the aqueous decoction of SWD [12]. Studies have shown that paeoniflorin balances immune cells and lymphocyte subpopulations through JAK2/STAT3 pathway [45]. The JAK/STAT signaling pathway is essential for the activation of extracellular cytokine receptors and subsequent signal transduction, which plays a critical role in cell proliferation, differentiation, organ development, immune homeostasis, and crosstalk with other pathways such as TGFβ, MAPK, Notch, PI3K/AKT/mTOR, and NF-κB. These interactions are crucial for regulating transcriptional programs in pluripotent and differentiated cells, immune function, and tumorigenesis [46, 47]. Given that the JAK/STAT signaling pathway is crucial for early hematopoiesis, our current study has conducted preliminary investigations on the effects of SWD on the JAK/STAT signaling pathway. Our findings demonstrated that SWD administration significantly reduced JAK1 levels in the spleen and BM, as well as decreased JAK2, STAT3, and IL6 levels, suggesting that SWD could mitigate immunosenescence induced by IR and might exert its effects by modulating the JAK/STAT signaling pathway to alleviate IR-induced inflammation and immune injury. However, further detailed studies are needed to fully elucidate the mechanisms by which SWD affects HSPCs and immune cells. More in-depth research on these mechanisms will be conducted in our subsequent studies and we will also explore different molecular targets, including: performing comprehensive studies to explore the impact of SWD on key signaling pathways, including but not limited to the JAK/STAT, MAPK, and PI3K/Akt/mTOR pathways; using advanced techniques such as RNA sequencing and proteomics to identify and validate molecular targets influenced by SWD; conducting functional assays to assess the biological outcomes of pathway modulation by SWD, such as cell proliferation, differentiation, and immune response assays and exploring the combined effects of SWD with other known modulators of these pathways to understand potential synergistic or antagonistic interactions. By undertaking these steps, a more comprehensive understanding of the mechanisms through which SWD exerts its effects on hematopoietic and immune cells will be provided. Besides, our investigation reveals an intriguing observation: the relative telomere length in BM cells treated with SWD exceeds that of the untreated controls. This finding raises the possibility that SWD might not only inhibit telomere shortening but also actively contribute to telomere elongation. To further explore this potential effect, we are conducting experiments using drosophila models to assess the impact of SWD on lifespan extension and to elucidate the underlying mechanisms. These ongoing studies will be comprehensively discussed in a subsequent publication.

In light of the promising results obtained from this study, the therapeutic potential of SWD in mitigating radiation-induced immune senescence merits further investigation for potential clinical applications. SWD, as a traditional Chinese medicine with a long history of safe use, possesses a unique combination of bioactive components that could synergistically target multiple pathways involved in the aging process and immune dysfunction. Our findings suggest that SWD may serve as a novel therapeutic strategy to mitigate the detrimental effects of radiation exposure, particularly in scenarios where radiation therapy is employed for cancer treatment or in the aftermath of radiation disasters. However, it is crucial to conduct rigorous preclinical and clinical trials to evaluate the safety, efficacy, and optimal dosing regimen of SWD in human subjects. After administering Siwu Tablets to SD rats via intragastric administration on the 45th day, 90th day, and 30 days after stopping the medication, histological examination revealed slight to mild extramedullary hematopoiesis in the spleen and enhanced erythroid hematopoiesis in the BM. This phenomenon, considered a pharmacological effect beyond the primary therapeutic action, suggests that the new Siwu Tablets have the potential to enhance hematopoiesis. The drug did not cause any damage to the organs, tissues, or systems throughout the body. Thirty days post-cessation, no accumulation or delayed toxic effects were observed in any of the groups. Through comprehensive analysis and evaluation, it was determined that the “safe usage dose without significant bodily harm” for the new Siwu Tablets could reach a dose level as 30 g/kg.

Future studies should also explore the possibility of combining SWD with other therapeutic modalities to enhance its therapeutic effects and broaden its clinical applications. At the doses tested in our experiments, no adverse effects were observed in mice. However, we recognize the importance of this issue and plan to conduct long-term toxicity studies in future research to thoroughly investigate any potential side effects of SWD. Ultimately, the translation of our findings into clinical practice has the potential to improve the quality of life for patients undergoing radiation therapy and mitigate the burden of radiation-induced health complications.

## Conclusion

This study demonstrated that continuous administration of SWD for 30 days following 8 weeks of total body irradiation at a dose of 6.0 Gy IR in C57 mice could attenuate radiation-induced senescence. SWD might mitigate the inflammatory response in the BM hematopoietic microenvironment by modulating the JAK/STAT signaling pathway; decelerate IR-induced hematopoietic senescence by preventing telomere shortening in BM cells and relieve IR-induced immune damage and immune senescence in mice by modulating HSPCs differentiation bias. The amelioration of hematopoietic and immunologic senescence was further confirmed at an overall level.

## Data Availability

Data will be made available on request.

## Abbreviations

BCA: Bicinchoninic acid assay
BM: Bone marrow
CAT: Catalase
CDKN2A/p16: Cyclin-dependent kinase inhibitor 2A
CLP: Common lymphoid progenitors
CMP: Common myeloid progenitors
DG: Dentate gyrus
ELISA: Enzyme linked immunosorbent assay
EO: Eosinophils
GMP: Granulocyte–macrophage progenitors
H2O2: Hydrogen peroxide
H&E: Hematoxylin–eosin staining
HGB: Hemoglobin
HPCs: Hematopoietic progenitor cells
HSCs: Hematopoietic stem cells
HSPCs: Hematopoietic stem/progenitor cells
IF: Immunofluorescence staining
IL-6: Interleukin-6
IOD: Integrated optical density
IR: Ionizing radiation
JAK1: Janus activated kinase 1
JAK2: Janus activated kinase 2
LSK: Lin − , Sca-1 + , c-Kit −
LYMPH: Lymphocytes
MDA: Malondialdehyde
MEP: Megakaryocytic-erythroid progenitors
MONO: Monocytes
MPP: Multipotent progenitor cells
NAD +: Beta-Nicotinamide adenine dinucleotide trihydrate
NEUT: Neutrophils
NK: Natural killer
NOP: Novel-object preference test
Nrf2: Nuclear factor erythroid 2-related factor 2
OFT: Open field test
qPCR: Quantitative real-time PCR
RBC: Red blood cells
RI: Recognition index
SASP: Age-related secretory phenotype
SOCS3: Cytokine signaling pathway inhibitor 3
STAT3: Signal transducer and activator of transcription 3
SWD: Siwu decoction
TCM: Traditional Chinese medicine
WBC: White blood cells
